# MuSCA: a multi-scale source–sink carbon allocation model to explore carbon allocation in plants. An application to static apple tree structures

**DOI:** 10.1093/aob/mcz122

**Published:** 2019-10-23

**Authors:** F Reyes, B Pallas, C Pradal, F Vaggi, D Zanotelli, M Tagliavini, D Gianelle, E Costes

**Affiliations:** 1 DAFNE, University of Tuscia, Viterbo, Italy; 2 DASB, CRI, Fondazione E. Mach, San Michele all’Adige, Italy; 3 Faculty of Science and Technology, Free University of Bozen-Bolzano, Bolzano, Italy; 4 AGAP, University of Montpellier, CIRAD, INRA, SupAgro, Montpellier, France; 5 CIRAD, UMR AGAP and Inria Zenith, Montpellier, France; 6 Amazon, Seattle, WA, USA

**Keywords:** MuSCA, multi-scale, carbon allocation, plant growth, tree growth, functional–structural plant modelling, FSPM, multi-scale tree graph, source, sink, apple, plant architecture

## Abstract

**Background and aims:**

Carbon allocation in plants is usually represented at a topological scale, specific to each model. This makes the results obtained with different models, and the impact of their scales of representation, difficult to compare. In this study, we developed a multi-scale carbon allocation model (MuSCA) that allows the use of different, user-defined, topological scales of a plant, and assessment of the impact of each spatial scale on simulated results and computation time.

**Methods:**

Model multi-scale consistency and behaviour were tested on three realistic apple tree structures. Carbon allocation was computed at five scales, spanning from the metamer (the finest scale, used as a reference) up to first-order branches, and for different values of a sap friction coefficient. Fruit dry mass increments were compared across spatial scales and with field data.

**Key Results:**

The model was able to represent effects of competition for carbon assimilates on fruit growth. Intermediate friction parameter values provided results that best fitted field data. Fruit growth simulated at the metamer scale differed of ~1 % in respect to results obtained at growth unit scale and up to 60 % in respect to first order branch and fruiting unit scales. Generally, the coarser the spatial scale the more predicted fruit growth diverged from the reference. Coherence in fruit growth across scales was also differentially impacted, depending on the tree structure considered. Decreasing the topological resolution reduced computation time by up to four orders of magnitude.

**Conclusions:**

MuSCA revealed that the topological scale has a major influence on the simulation of carbon allocation. This suggests that the scale should be a factor that is carefully evaluated when using a carbon allocation model, or when comparing results produced by different models. Finally, with MuSCA, trade-off between computation time and prediction accuracy can be evaluated by changing topological scales.

## INTRODUCTION

Carbon (C) allocation is the process by which C assimilated by leaves or stored in the form of carbohydrates is transferred, via the conductive vessels (or phloem tissue), to other plant parts, where it is used primarily for respiration and growth. It is generally accepted that the phloem sap moves as a consequence of an osmotically generated pressure gradient, as described by the Münch theory (Münch, 1930). Gradients are generated by the differences in the concentrations of C assimilates between C sources (mainly leaves, where C assimilates are loaded into the phloem) and sinks (where C assimilates are unloaded from the phloem). The Münch theory has been refined over time (e.g. with the introduction of the leakage–retrieval mechanism) (Thorpe *et al.*, 2005), and complementary hypotheses have been proposed to explain the lack of fit with experimental evidence (e.g. sieve tubes decomposed in shorter, overlapping components, at the edge of which solutes are transported at the expense of internal energy) (De Schepper *et al.*, 2013). The underlying principles, however, are still considered valid. Thus, the C allocation process is thought to depend primarily on the amount and distribution of the available C supplies, on demands along the plant structure and on the possibility that C supplies flow via the phloem (Ryan and Asao, 2014). In models of small plants, however, the simplifying assumption that distance has no effect on C availability or phloem osmotic potential (known as the ‘common assimilate pool’) can be made (Heuvelink, 1995; Guo *et al.*, 2006; Luquet *et al.*, 2006; De Swaef *et al.*, 2013).

When plant topology increases in complexity, the modelling approaches to C allocation present in the literature can be organized in four, partly overlapping, categories (Thornley and Johnson, 1990; Lacointe, 2000; Le Roux *et al.*, 2001; Génard *et al.*, 2008): models based on empirical relationships between plant parts and/or the environment; teleonomic models representing the plant as moving towards a specific state defined *a priori*; source–sink-driven models, in which different plant components are supposed to attract C assimilates with different strengths; and transport resistance and biochemical models, which represent phloem transport as starting from osmotic gradients and biochemical conversions. The most mechanistic models, which represent osmotic flows as starting from osmotic gradients, imply complex formalizations and highly detailed plant descriptions and have therefore received relatively limited attention (Bancal, 2002). The modest mathematical complexity of source–sink models has led to them becoming the focus of a much larger number of studies. In all cases the representation of C allocation on large tree structures described at high resolution remains computationally intractable (Balandier *et al.*, 2000; Bancal, 2002).

Each model represents the plant structures, and the process of C allocation, at a specific scale. The choice of scale may be driven by management or research needs, by the computation time needed for the simulations and by the resolution at which the calibration and testing data were obtained. Such scales range from the individual metamer (Allen *et al.*, 2005) to collections of metamers that, grouped according to criteria such as their age, position or organ type (Kang *et al.*, 2008), constitute larger portions of a tree structure, such as branches, main axis or even whole plant compartments (Lakso and Johnson, 1990; Kang *et al.*, 2008).

By way of example, the L-PEACH model (Grossman and DeJong, 1994; Allen *et al.*, 2005) is a reference for completeness of the described processes and represents tree growth over multiple years. This uses a transport resistance analogy to mimic C transport at the metamer (M) scale (internode, leaf, fruit). The SIMWAL model represents the growth of a young walnut tree across several years. This is organized in axes, which are divided into growth units (GUs, the scale of C allocation), themselves composed of internodes and nodes (Balandier *et al.*, 2000). A GU is an organization level typical of plants with rhythmic growth. It corresponds to a portion of axis developed without interruption during an elongation period, and can be visually identified as bounded by two zones of scales that had protected the apical meristem in the bud during resting period. The QualiTree model represents growth and quality of fruits on a peach tree structure, at the fruiting unit (FU) scale, during a single growth season (Lescourret *et al.*, 2011; Pallas *et al.*, 2016). An FU includes the set of shoots born from a common section of 1-year-old wood. In this model all the different types of organs in an FU are considered similar with respect to assimilate exchange, which depends on their respective distances only.

The computation of C allocation by a model is affected by both the formalism used to describe the physiological processes and the discretization of the plant. Because of this, identifying what causes differences in results produced by different models can be difficult.

The present work proposes a new, source-sink, multi-scale C allocation model (named MuSCA), whose aim is to allow the flexible definition of topological plant scales concomitant with the simulation of C allocation. The model relies on the use of a multi-scale tree graph (MTG) (Godin and Caraglio, 1998), a formalism inspired by the observation of the multi-scale organization of plant structures (Barthélémy, 1991; Barthélémy and Caraglio, 2007; Balduzzi *et al.*, 2017). An MTG allows the topological description of a plant at multiple, nested, spatial scales on the same graph. In addition to the connections and boundaries of the described topological scales, intensive (e.g. organ type) or extensive (e.g. geometrical features) properties can be assigned to the individual plant component at any scale. MTGs have been previously used in plant architecture analyses (Costes *et al.*, 2003) and in radiation interception (Da Silva *et al.*, 2014*a*), as well as to simulate physiological processes (Fournier *et al.*, 2010; Ndour *et al.*, 2017; Barillot *et al.*, 2018) or model plant pathosystems (Garin *et al*., 2014, 2018; Robert *et al.*, 2018) (albeit by using a single scale at a time).

In this paper we provide an overview of the MuSCA model, define its scope and inputs, and present the formalisms used to compute C allocation and to move across scales. The model is calibrated for the apple tree (*Malus* × *domestica*) ‘Fuji’, for C demands. It is linked to a radiative model for the estimation of C assimilates by leaves. The model is then applied to three contrasted apple tree structures and with different values of an empirical sap friction parameter, and the effects of competition for C assimilates on fruit growth are analysed. Field observations are then compared with the fruit growth distribution simulated on one tree structure to retain the sap friction parameter values that might best represent sap flow dynamics. The ability of the multi-scale formalism to produce similar results across scales and the influence of the scale of representation on the simulated C allocation are analysed. Finally, the trade-off between computational efficiency and accuracy are discussed with respect to the possible interactions between specific tree structure and the topological scale used.

## MATERIALS AND METHODS

### Model description

#### Overview.

**Fig. 1.**
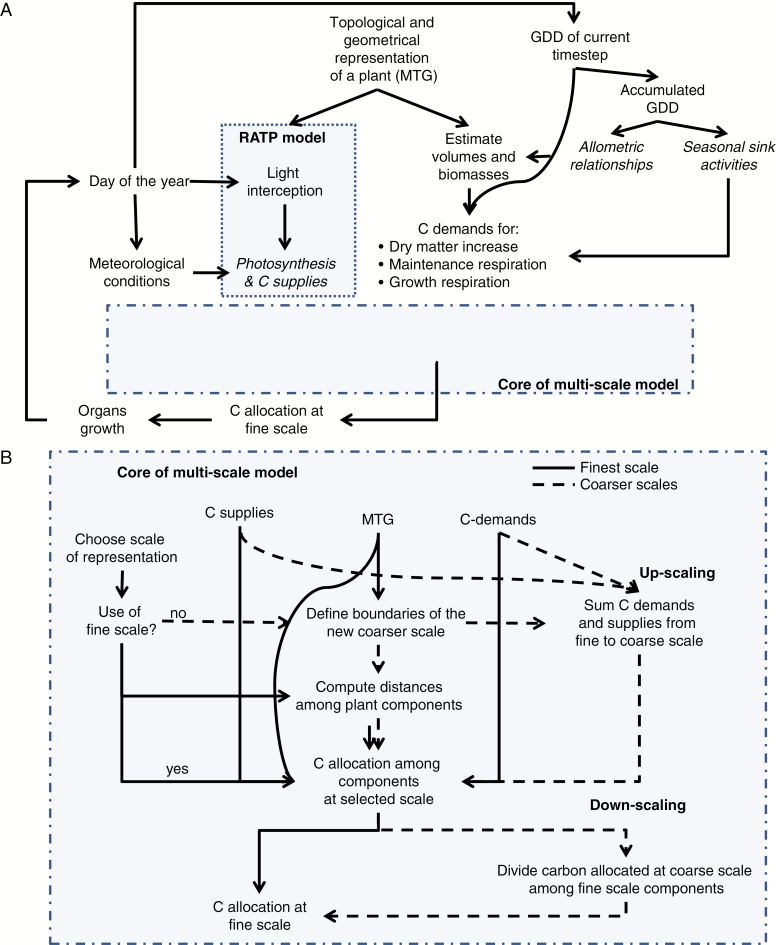
Conceptual workflow of the model: (A) general framework for the application of the model; (B) the core of MuSCA. (A) A multi-scale tree graph representing a plant structure at a given developmental stage is provided. This is used, together with the growing degree days (GDD), allometric relationships and plant geometrical information to compute initial volumes and biomasses of plant components at metamer (M) scale. Carbon demands are estimated at M scale by making use of species-specific seasonal sink activities, biomasses and GDD at the current time step. The position of individual leaves on the plant structure is used by the radiative model RATP to compute light interception and, together with meteorological conditions, individual leaf photosynthesis. (B) The workflow follows different paths depending on the selected scale: M scale (solid lines) and coarser scales (dashed lines). The scale at which the plant is represented determines up- and down-scaling processes. If a coarse scale is chosen, C demands and supplies are up-scaled. Carbon allocation is then computed among the plant components at the chosen scale. After allocation, if the selected scale was a coarse one, the C allocated to each component is divided among its constituent elements. Biomass of individual elements is eventually updated prior to moving to the next time-step. Steps containing species-specific parameters are shown in *italics*.

MuSCA is a generic, functional–structural plant model (FSPM) (Vos *et al.*, 2010) representing the C allocation and organ growth of a plant at different, user-defined, spatial scales, while taking into account competition and distances between C sinks and supplies present in the plant (Fig. 1). The model has a modular architecture and each module is generalized so that only a few of them are specific to a given cultivar or species (Tables 1 and 2). The model is integrated in the open-source OpenAlea environment (Pradal *et al*., 2008, 2015) and implemented in the Python language. The model re-uses existing OpenAlea components, such as the MTG dynamic data structure (Godin and Caraglio 1998; https://github.com/openalea/mtg), used to represent the multi-scale topology of plant structures, the PlantGL library (Pradal *et al.*, 2009) used to represent the 3-D geometry, and the RATP (radiation, absorption, transpiration and photosynthesis) radiative model (Sinoquet *et al.*, 2001). The MuSCA model simulates biomass accumulation over a single vegetative season.

**Table 1.** Model parameters and equation for estimation of biomasses

Parameter	Description	Value/range	Unit
dry_mass_to_C_mass	Ratio to convert carbon assimilated into biomass uptake	2.105263158	–
wood_density	Old wood density	700	kg m^−3^
spec_leaf_surface	Per unit surface leaf dry mass	0.08	kg m^−2^
dry_to_fresh_DWratio	Ratio to convert apple fruit fresh to dry weight	0.15	–
shoot_to_root_growth	Vegetative shoot to root biomass ratio	4.5	–
Generalized linear model to estimate shoot biomass (g) from length (cm) and thermal time (°C)			
Dry weight = exp(*a* + *b* × log(length) + *c* × GDD + *d* × length + *e* × log (length × GDD))			
*a*	−3.073409537		
*b*	0.706724497		
*c*	5.78E−05		
*d*	0.020013707		
*e*	9.81E−05		

**Table 2.** Model parameters and equations for estimation of C demand for dry matter increase

Parameter	Description	Value/range	Unit
trunk_activity	Sink activity for old wood	0.000031	g × g^−1^ × C^−1^
leaf_activity	Range of values for leaf activity (from Look Up Table)	0 to 1.9 × 10^−3^	g × g^−1^ × C^−1^
Gompertz equation for fruits and shoots			
Dry weight = *a* × exp(*b* × exp(*c* × GDD))			
Normalized derivative			
Relative growth rate =			
(*a* × *b* × *c* × exp(*b* × (exp(*c* × GDD)) + *c* × GDD))/(*a* × exp(*b* × exp(*c* × GDD)))			
Parameter	Fruit	Proleptic shoot	
*a*	63.703	2.406	
*b*	−4.896	−2.84	
*c*	−0.00104	−0.00165	
GDD, growing degree days after bloom, after cut-off of base temperature of 4.5 °C.			

#### Input plant structures and creation of new scales.

The input plant structures of MuSCA are MTGs where individual plant components are characterized by qualitative or quantitative information. Qualitative information refers to the organ type or the type of connection between vertex and parent (i.e. branching or succession). Quantitative information may include parameters such as the geometry or age of a plant component. This information can be used to create criteria defining the membership of individual plant components in larger groups of adjacent plant components.

#### Movement of carbon.

The movement of the available C (*F*_*ij*_), from a C supply (*i*) to a C sink (*j*), is represented as a function of the C available in the supply (ACP_*i*_) and the sink C demand (Demand_*j*_) (eqn 1a), and is inversely related to the distance (dist) between source and sink and to a sap friction parameter (*h*) (eqn 1b), along the plant topology. The *h* factor is an empirical friction parameter, proposed in previous source–sink C allocation models (Balandier *et al.*, 2000; Lescourret *et al.*, 2011). Larger values of this parameter amplify the effects of distance, resulting in relatively large amounts of C allocated next to the source, while a value of zero implies no effect of distance. This equation is inspired by a previously defined equation (SIMWAL model; Balandier *et al.*, 2000).

$$F_{ij}=\frac{{\text{Demand}}_{j}\text{ ​​}\;\text{​​ }\times \text{ ​​}\;\text{​​ }f\left({\text{dist}}_{ij},h\right)\text{ ​​}\;\text{​​ }\times \text{ ​​}\;\text{​​}{\text{ ACP}}_{i}}{\sum_{k\;=\;1}^{n}{\text{Demand}}_{k}\text{ ​​}\;\text{​​ }\times \text{ ​​}\;\text{​​ }f(dist_{ik},h)}$$ 1a

$$\begin{array}{l} f(dist_{ij},h)=\frac{\;\;\;1}{{\left(1\;\;\;+\;\;\;dist_{ik}\right)}^{h}} \end{array}$$ 1b

If in excess, the C allocated to a plant component will just fulfil its C demand, while the excess will be stored in a reserve pool for this compartment and be considered as a supply provided by that component at the following time-step (Balandier *et al.*, 2000). However, since simulations in this study lasted for only 1 d, this phenomenon never occurred.

The C allocated to a topological element is eventually divided among the different organs that constitute it (at the metamer scale: fruit, leaf, internode), proportionally to their individual C demand.

#### Calculation of distances.

A representation of the C allocation process coherent at multiple topological scales requires the definition of an equation for the calculation of distances, independent of the scale of representation.

Modelling C allocation is computationally demanding as, in a source–sink formulation, it requires computation of the distance between all pairs of C sinks and sources in the tree. In an MTG, sources and sinks are represented by vertices in a tree graph. The complexity of computing the distance between all pairs is quadratic, i.e. requires around *n*^2^ operations for a tree of *n* vertices. We thus designed an algorithm, based on the multi-scale organization of plants, that makes it possible to reduce the number of operations by reducing the number of independent plant vertices that must be compared.

Each MTG vertex (*i*) is characterized, among other properties, by 3-D coordinates (basal and top), the possibility of having a parent (named *i*-1), and one or multiple sons (the vertex *i* is the son of its parent). In an MTG, a vertex at a coarser scale (*I*, called complex) is composed of one or more vertices at the finer scale (called components of *I*), as in the case of a shoot composed of several internodes.

**Fig. 2.**
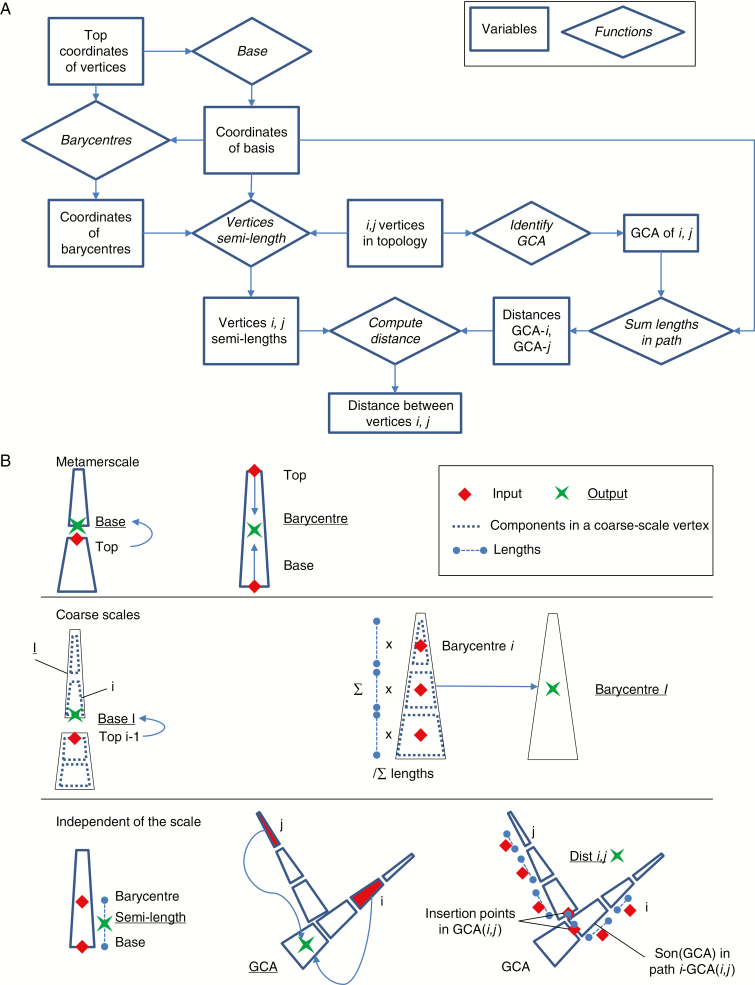
Workflow and graphical representation of the calculation of distances. (A) Workflow for calculation of the distance between two vertices, *i* and *j*. Coordinates of vertices are used to compute coordinates of bases and barycentres for the whole topology. Given two vertices *i*, *j*, their distance is calculated in three steps: the greatest common ancestor (GCA) of *i*, *j* is identified; an iterative process sums the length of the vertices connecting GCA to *i* and *j*; the semi-lengths of *i* and *j* are also summed. (B) (Top) Graphical representation of the: calculation of coordinates of bases and barycentres at (above) metamer (M) scale. The base of a vertex corresponds to the top of its parent; coordinates of the barycentre of a vertex are calculated from those of its top and base. (Middle) The base of a coarse-scale vertex (capital *I*) corresponds to the base of its first component (lower-case *i*), which is inherited from the top of its parent at M scale (lower-case *i*-1); coordinates of the barycentre of a coarse-scale vertex are obtained as the mean of the coordinates of its own components at M scale, weighted by their lengths. (Bottom) Calculation of the semi-length of a vertex, identification of GCA and computation of distances along a path connecting two vertices *i*, *j*. Data used as input of calculations are indicated by red rhombi and are connected by arrows to the respective outputs, represented by green crosses.

The distance (dist_*i,j*_) between two vertices (*i*, *j*) in an MTG depends on the sequence of vertices that connect *i* and *j* (called the topological path). It corresponds to the sum of (1) the semi-lengths of the extremities *i* and *j*, and (2) the length of the topological path between the bases of *i* and *j* (eqns 2a and 2b; Fig. 2). The semi-length of an element at different scales is computed as the distance between its base and barycentre.

In order to assess the length of this path, it is necessary to identify the greatest common ancestor (GCA) of vertices *i* and *j* (Fig. 2). Given the two paths connecting the tree root to *i* and *j*, the GCA of vertex *i* and vertex *j*, GCA(*i*,*j*), is identified as the vertex that is contained in both paths and that is the closest to *i* and *j*. The topological path between *i* and *j* is the union of the vertices between *i* and GCA(*i*,*j*) and the path between *j* and GCA(*i*,*j*) with GCA(*i*,*j*) (Fig. 2). The length of the path (*dist*_*i,j*_) is computed as the sum of the length of each of its internal elements plus the semi-lengths of its extremities, minus twice the length of the GCA (Fig. 2, eqn 2a). In addition, the distance between the basis of the first two elements of the path, which are directly inserted in the GCA(*i*,*j*) and are not connected with each other, is added (eqn 2a). This is the case at coarser scale when different ramifications are connected to the trunk at different location. When the GCA is either *i* or *j*, the length is just the sum of the length of all its internal elements plus the semi-length of its extremities (eqn 2b).

$$\begin{array}{ll} dist_{i,j}=\; & semi-length_{i}+dist_{i,GCA}+dist_{j,GCA}\\ & +semi-length_{j}-2length(GCA)\\ & +dist(base_{son(CGA)i},base_{son(CGA)\;j}) \end{array}$$ 2a

where son(GCA)i(resp. *j*) is the first descendant in the path (*i*,GCA(*i*,*j*))(resp. *j*).

If GCA is *i* or *j*,

$$\begin{array}{l} dist_{i,j}=dist_{i-1,j-1}+semi-length_{i}+semi-length_{j} \end{array}$$ (2b)

The calculation of the spatial coordinates of the basis and barycentres depends on the scale (Fig. 2B). At M scale, the basal coordinates of a vertex correspond to the top coordinates of its *parent* vertex (eqn 3a). For the basal vertex of the MTG, the only vertex without a parent, coordinates are stored in the complex of the same vertex. Barycentre coordinates are obtained as the mean of its top and base coordinates (eqn 3b).

At coarser scales, the basal coordinates of a vertex *I* are the basal coordinates of its first component at the metamer scale, i.e. the one whose parent does not belong to *I* (eqn 3c). These basal coordinates are inherited from top coordinates of its parent (eqn 3a).

Regarding the coordinates of the barycentre of a coarse-scale vertex, these are calculated as the mean of the coordinates of the barycentres of its components, weighted by their individual lengths (as in eqn 3d for the *x* coordinate).

At metamer scale:

$$\begin{array}{l} base_{i}=top_{parent(i)} \end{array}$$ (3a)

$$\begin{array}{l} barycentre_{X,i}=\frac{top_{X,i}\;+\;\;\;base_{X}}{2} \end{array}$$ (3b)

At coarse scales:

∀ *i* ⊆ *I* | parent(*i*) not ⊆ *I*:

$$\begin{array}{l} base_{I}=top_{parent(i)} \end{array}$$ (3c)

$$\begin{array}{l} barycentre_{X,\;\;\;I}=\frac{\overset{}{\sum }barycentre_{X,\;\;\;i}\;\;\;\times \;\;\;length_{i}}{\overset{}{\sum }length_{i}} \end{array}$$ (3d)

#### Up- and down-scaling.

In MuSCA, some of the plant properties available at one scale are used to provide a description of the same properties at a coarser (up-scaling) or finer (down-scaling) scale. This is done in simulations running at coarse scales, before and after the calculation of C flows, respectively, by calling up- and down-scaling functions (except for the initial calculation of biomasses; see Supplementary Data File S1). The up-scaling of properties such as biomass, C supplies and demands of plant parts is simply done by summing the property values stored in all the vertices that are contained within the topological boundaries of the coarser-scale component. Conversely, down-scaling the same properties from a coarse-scale component to its constituting elements requires some assumptions. In particular, when the C allocated to a coarse-scale component is down-scaled, it is assigned proportionally to the relative C demands of its constituent elements.

### Application to realistic tree structures: the case of apple (*Malus × domestica*)

In order to assess the ability of the model to simulate similar growths across multiple scales, to represent competition for C assimilates and the effect of the scale on computation time, we applied MuSCA to different tree structures of apple trees, represented at multiple topological scales. These were MTGs of three apple trees simulated by the MAppleT architectural model (Costes *et al.*, 2008). MAppleT combines Markovian models for the simulation of GU succession and branching with a biomechanical model for axis bending. Along GUs, individual leaf area was simulated based on a logistic function and assuming different final values of leaf areas for the preformed (seven leaves) and neoformed parts of the GU (Da Silva *et al.*, 2014*a*). This model was previously calibrated for the ‘Fuji’ cultivar (Costes *et al.*, 2008) and so it could be used to generate the one 4-year-old ‘Fuji’ tree that was used in this study. Moreover, two other trees were simulated by initiating simulations using two different sequences of lateral types along the trunk observed on trees originated from a biparental population, as previously proposed by Da Silva *et al.* (2014*b*). These two trees were selected within the population in order to test our C allocation model on trees displaying contrasted architectures. Simulations were run for each individual tree on a single day, so that the direct effect of changing the topological scale could be analysed while excluding possible retroactions and cumulative effects (such as the re-use of C delivered in excess to a plant component). A partly cloudy day in late June (day of the year = 182) was chosen to drive photosynthesis. This made it possible on the one for hand C-limiting conditions to occur (the use of very sunny days resulted in C allocation generally satisfying the whole-fruit C demand), and on the other hand to represent trees in which shoot elongation, and thus the creation of new internodes, had ended. Initial fruit dry weight was set to be identical for all fruits and equal to 8 g (Reyes *et al.*, 2016).

For the application of the C allocation model to tree structures, we developed a few modules containing some species-specific parameters and allometric relationships (Tables 1 and 2). These were needed firstly to convert the geometrical descriptions of the trees contained in the MTGs into values of biomass (see Supplementary Data File S1), and then to estimate the values of C demands for dry-matter accumulation (see section Source and sink strengths).

#### Source and sink strengths.

In MuSCA, the amount of C assimilates available from photosynthesis in individual leaves is estimated with the radiative model RATP (Sinoquet *et al.*, 2001) integrated within the OpenAlea platform and running on MTG structures (see also Supplementary Data File S1). In RATP the tree structure is first discretized into voxels of user-defined size. The voxel-specific mean leaf area density (turbid medium assumption) is calculated based on the 3-D representation of the plant in space. This is then used to compute the direct and diffused photosynthetically active radiation and near infrared radiation light intercepted in each voxel, and its related photosynthesis, every 30 min, across the whole day. The C assimilation estimated per leaf unit surface is first associated with each leaf and integrated over the whole day, and then converted into dry matter uptake per leaf per day. In this study, a previous calibration of RATP for apple trees, ‘Fuji’ cultivar (Massonnet *et al.*, 2006), was used.

Sink strengths were calculated as the sum of the C demands for dry matter accumulation into the plant structure and the C lost by respiration (Table 2). The first term was assumed to follow maximum potential relative growth rates. Maximum potential growth curves were obtained by fitting thermal-time-dependent Gompertz functions to organ-type-specific maximum dry weights (Grossman and DeJong, 1995) of proleptic and epicormic shoots, and fruits of ‘Fuji’ apple trees growing in conditions where competition for carbohydrates was minimized (Reyes *et al.*, 2016). Their normalized derivatives (Hunt, 1982; Grossman and DeJong, 1995) represent the seasonal patterns of organ-type-specific activities (or maximum potential relative growth rates). Regarding the old wood, the slope of a linear relationship fitted through the logarithm of the relative growth rate of the old wood biomass versus growing degree days (GDDs) obtained by Reyes *et al.* (2016) was used. Values of sink activities of the current days were then used, together with organ dry weight and GDD, to estimate the C demand for dry matter accumulation ($DM\_Demand_{j}$*) of the same day (eqn 4) (*Marcelis, 1996*) at the metamer scale.*

$$\begin{array}{ll} DM\_Demand_{j}=\; & sink\;dry\;weight\;\\ & \times \;sink\;activity{\left(GDD\right)}_{organtype}\;\\ & \times \;GDD_{day} \end{array}$$ (4)

Respiration was split into maintenance and growth components as proposed by Lescourret *et al.* (2011) and Pallas *et al.* (2016). The maintenance component was modelled as proportional to dry mass, a Q10 law and a maintenance respiration parameter. Growth respiration was considered to be proportional to the growth respiration coefficient and to the sum of the C demanded for maintenance respiration and for the dry matter increase. Parameter values provided by Pallas *et al.* (2016) were used.

#### Definition of topological scales.

**Fig. 3.**
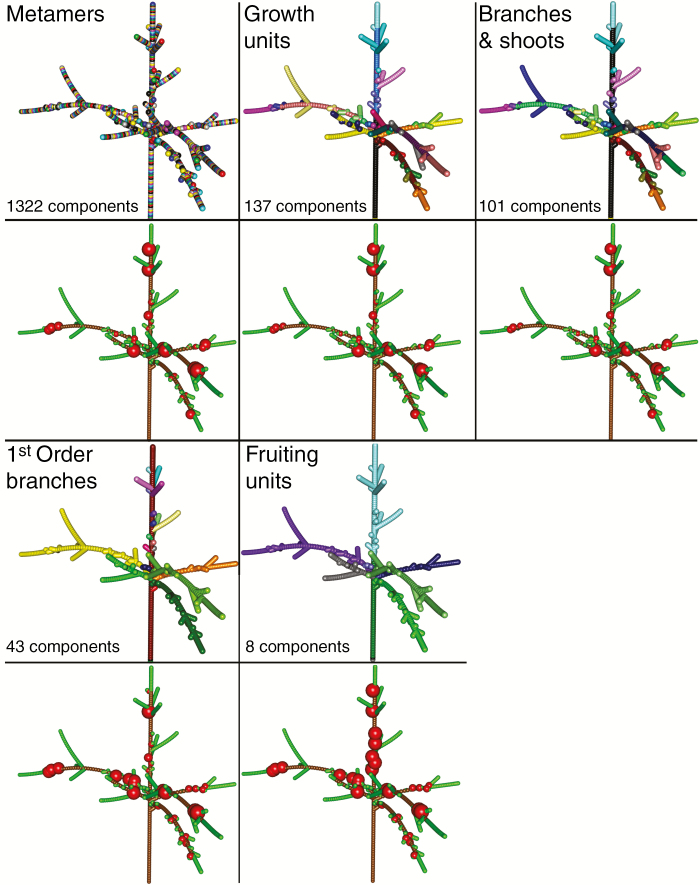
Simulation of C allocation and fruit growth on a tree structure represented at different spatial scales during one day. (First and third rows) Tree parts represented with the same colour belong to the same scale, i.e. trees with a higher number of colours have a higher number of components. The number of components at each scale is indicated in the corresponding lower left corner. (Second and fourth rows) The volume of the represented spheres is proportional to the normalized increment in fruit dry weight within the tree. Simulations were run with a friction parameter equal to 8.

Five biologically relevant scales of representation of trees were used. Two of them are commonly used in MTGs (metamer and GU) and three are newly defined (trunk, branches and shoots; first-order branch and inter-branches; fruiting unit) (Fig. 3). New scales were defined by setting criteria for the identification of edges, along the plant topology, among which all elements could be considered as belonging to the same group. The finest scale considered was the metamer (or phytomer), which is composed of a node and its leaf(ves) and axillary bud(s) plus the subtending internode. This scale was considered as a reference for the representation of C allocation in this study. The second scale corresponded to the GU (Barthélémy, 1991). The third scale corresponded to the discretization of the tree in the main trunk, first-order branches originating from it and the leafy shoots [trunk, branches and shoots (TBS)]. The fourth scale corresponded to first-order branches originating from the main trunk, but without considering the individual shoots separately (BR1). The fifth scale corresponded to the FU. In all scales, the root was considered as a compartment in itself.

#### Simulation of fruit growth.

We ran simulations for the three apple tree structures represented at the five topological scales defined above. In order to identify a biologically sensible value of the friction parameter (*h*), this was varied across a range of almost two orders of magnitude (0.5–16).

We tested the model’s ability to simulate competition for the available C among tree organs at M scale. This was done by analysing the growth of individual fruits in relation to both the fruit load and the C assimilated in the surroundings of the analysed fruit. In particular, a neighbourhood of the analysed fruit is defined as the set of metamers for which the geometrical distance between their barycentre and the barycentre of the metamer containing the fruit is lower than a given threshold.

Considering a growing fruit, the ratio between the amount of C assimilated and the number of fruits present in its neighbourhood represents a comfort index with respect to the availability of C for fruit growth and the occurrence of competition with other fruits. Thus, the correlation between this ratio and the simulated growth of individual fruits belonging to the same neighbourhood illustrates whether fruit growth is affected by the competition for C assimilates occurring within the area around the analysed fruit. Based on these considerations, the values of C assimilated and the number of other fruits in the surroundings of individual fruits present on each tree was calculated for neighbourhoods of incremental radii (radii of 5, 15, 25, 35, 45, 55, 65, 75, 95, 115 and 135 cm) and for different friction parameters. Then, a correlation between the ratio (C assimilated/number of fruits) and the simulated growth of individual fruits belonging to the same neighbourhood was calculated for each radius and friction parameter value. Significance of the correlations was then adjusted for multiple comparisons by using Bonferroni correction (i.e. dividing the significance level of the test, *P* = 0.05, by the number of correlations for which significance was tested: 11 distances).

Biological relevance was also tested by comparing the simulated growth with the ‘Fuji’ tree structure and harvested fruit size distribution with field trees of the same age (4 years) and cultivar. Based on the assumption that fruit weight obtained in early growth stages is correlated to fruit weight at harvest (Stanley *et al.*, 2000), we compared the distributions of the normalized values of the C allocated in our simulations with fruit weight measured at harvest (E. Costes, unpubl. data). Similarity among distributions was also assessed in terms of root mean square error (RMSE) between distribution counts. Based on these results, a narrower range of friction parameter values was identified as more biologically relevant and used in further analysis.

The coherence of simulations across scales was tested by correlating fruit growth obtained at each coarse scale with that at M scale, used as a reference. Results obtained for equal friction parameters and the same tree structure were considered together. For each coarse-scale component, the average of fruit growth obtained at M scale within the boundaries of the coarse-scale component was computed. Thus, a one-to-one comparison was possible between mean growth at M and other (coarser) scales. Deviations between M and the other scales were assessed visually on correlation plots and by means of the coefficient of variation (CV) of the RMSE.

The effect of using different topological scales on the number of plant components and on simulation time was also analysed. All simulations were run on a computer equipped with an Intel i7-6700HQ 2.59 Ghz CPU (8 Gb RAM; Windows 10 Home, 64 bit).

## RESULTS

### Testing physiological assumptions

Relative growth rate (RGR) of shoots in the ‘Fuji’ tree structure at M scale increased with the value of the friction parameter (*h*), while for fruits and old wood it increased as *h* values increased from 0.25 to 4 and 2, respectively, and then decreased as *h* increased to 16 (Table 3). In other words, the higher the value of the friction parameter the higher was the growth occurring close to the C source. The RGRs of old wood and shoots were between normal growth observed in the field and maximum potential growth data used for the calibration of sink demands (between 5.3 × 10^−3^ and 3.1 × 10^−2^ mg g^−1^ for the old wood and between 0.4 and 1.2 mg g^−1^ for shoots; Reyes *et al.*, 2016). Conversely, fruit growth was between 35 and 51 % lower with respect to the fruit growth observed in normal field conditions (1.9 and 2.8 mg g^−1^ for fruits).

**Table 3.** Relative growth rate at M scale for different friction parameter values (*h*) in the ‘Fuji’ apple cultivar.

Relative growth rate (mg g^−1^ dd^−1^)			
*h*	Old wood	Shoots	Fruits
0.25	0.016	0.50	1.19
0.5	0.016	0.50	1.20
1	0.018	0.50	1.20
2	0.019	0.51	1.22
4	0.018	0.54	1.23
8	0.015	0.63	1.17
16	0.009	0.63	0.93

**Fig. 4.**
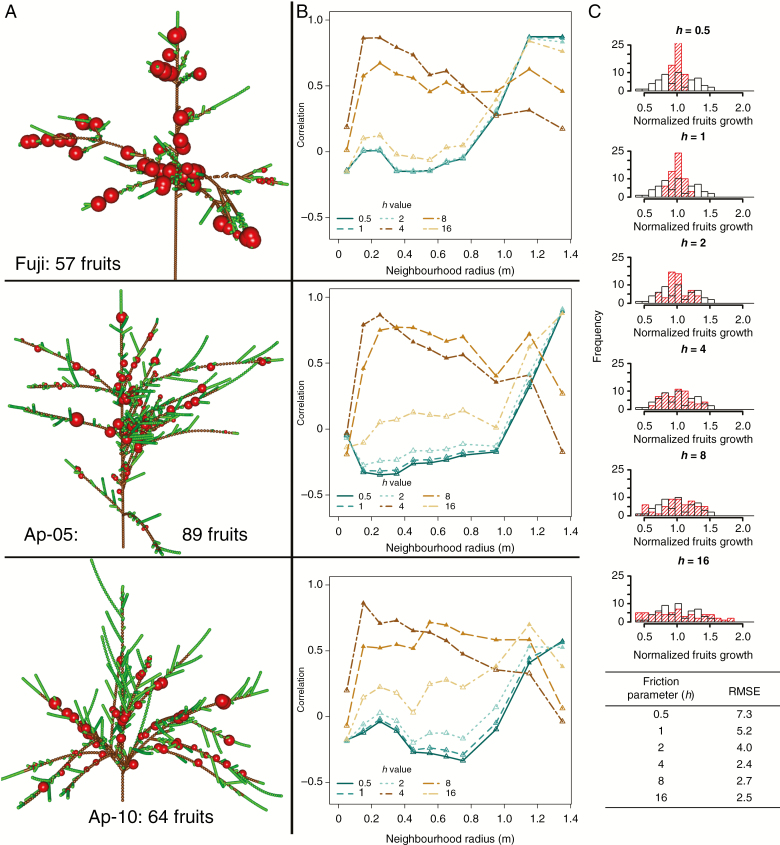
Physiological responses in simulations at M scale with respect to different tree structures and friction parameters (*h*). (A) 3-D representation of the tree structures with fruit growth (obtained with *h* = 8). (B) Correlation between individual fruit growth and the ratio between C assimilated and number of fruits evaluated in neighbourhoods of increasing radius. (C) Distributions of normalized simulated fruit growth (red, coloured bars) superposed on normalized fruit dry weight measured at harvest (open bars), in the 4-year-old simulated and observed ‘Fuji’ trees for different *h* values. The RMSE between distribution counts for different *h* values is provided. The upper parts of the histograms for *h* < 2 are cut off in order to allow better visualization of results on the vertical axis.

Simulation results at M scale show the impact of the friction parameter (*h*) in determining the area around an individual fruit within which this competes for C assimilates with other fruits (Fig. 4A, B). As shown by the significant correlations among fruit growth and comfort index (ratio between C assimilated and number of fruits), the neighbourhood within which fruit growth is affected by competition for C is relatively large (>0.9 m) for small values of the friction parameter (0.5–4). This means that the within-tree variability in fruit growth is mostly related to C sources located far from it. In other words, a large part of the tree structure affects the growth of each individual fruit. Conversely, for high friction parameter values (8, 16), fruit growth is affected mainly by the C provided by close leaves and possibly the competition with neighbouring fruits (neighbourhood <0.9 m) (Fig. 4B).

The normalized fruit dry weight simulated for the 4-year-old ‘Fuji’ tree was compared with that of fruits measured at harvest in two similar trees in order to evaluate what range of the friction parameter best fitted the observed fruit size distribution (Fig. 4C). The lowest RMSE values were obtained with *h* values between 4 and 16. High values, however, produced skewed distributions with variability larger than that obtained in the field (*h* = 16). Conversely, for low *h* values (0.5, 1) distributions had consistently lower variability than in the field. Similar variability and low RMSE values were obtained with *h* values of 4 and 8.

### Carbon allocation at multiple scales

Changing the scale of representation of the tree from M to coarser scales sharply decreased the number of represented topological components of the tree structures. Trees at GU, TBS, BR1 and FU scales contained about 12.7, 8.6, 1.6 and 1.2 %, respectively, of the elements they had at M scale (Table 4).

**Table 4.** Number of components included in tree structures at different scales

Tree structure	Scale	Number of components	%	Computation time (min)	%
‘Fuji’	M	3355	100	175.6	100.0
	GU	521	15.5	1.0	0.6
	TBS	320	9.5	0.6	0.4
	BR1	29	0.9	0.4	0.2
	FU	67	2	0.4	0.2
Ap-05	M	4579	100	313.8	100.0
	GU	535	11.7	1.5	0.5
	TBS	402	8.8	1.1	0.4
	BR1	115	2.5	0.7	0.2
	FU	36	0.8	0.7	0.2
Ap-10	M	4083	100	246.4	100.0
	GU	449	11	1.1	0.4
	TBS	302	7.4	0.8	0.3
	BR1	59	1.4	0.6	0.2
	FU	36	0.9	0.6	0.2

**Fig. 5.**
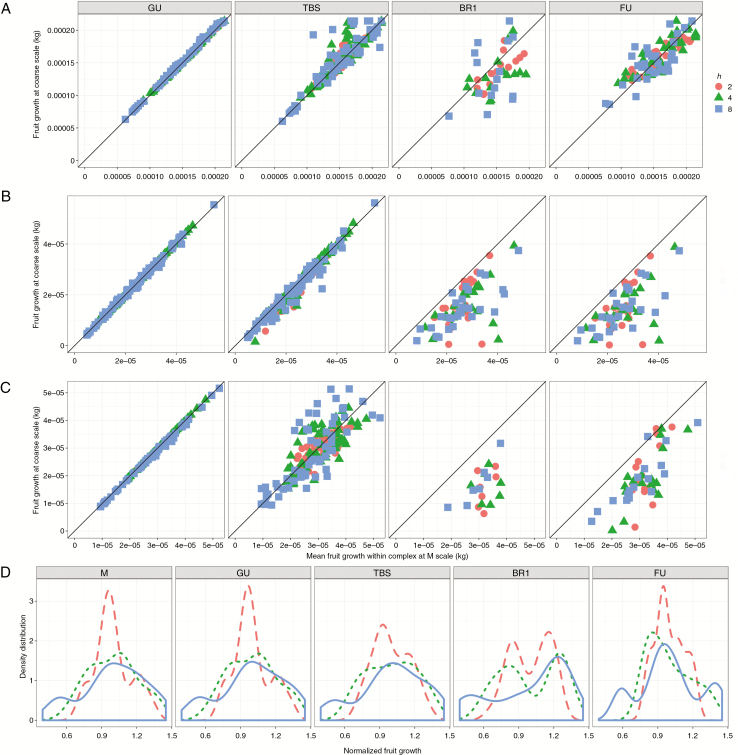
Mean daily fruit dry weight increment and its distribution (in ‘Fuji’ apple) depending on topological scale (from left to right), tree structure (from top to bottom) and friction parameter *h* (coloured symbols). Correlation between C allocated to fruits per day at the selected coarse scale (*y* axis) versus C allocated to fruits per day at M scale and averaged for all metamers belonging to the same component at coarser scale (*x* axis) is shown for (A) ‘Fuji’, (B) Ap-05 and (C) Ap-10. (D) Fruit growth distribution in the ‘Fuji’ tree structure (dashed, *h* = 2; dotted, *h* = 4; solid, *h* = 8).

**Fig. 6.**
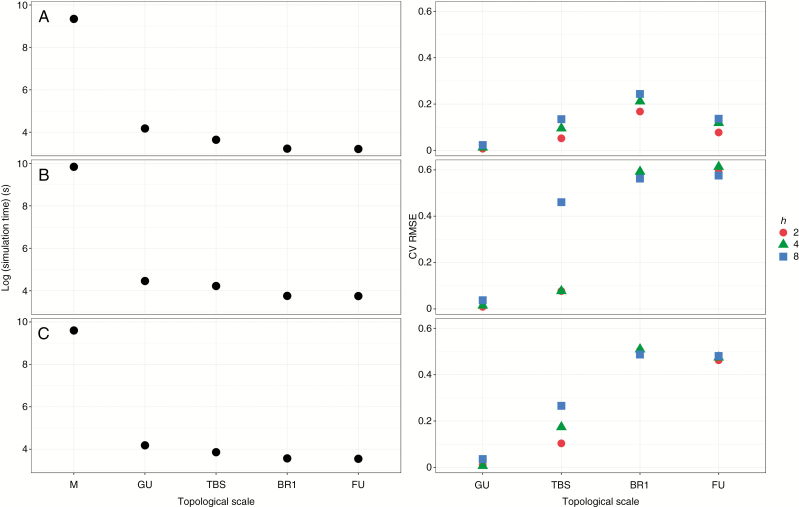
Trade-off between computation time and prediction error. The logarithm of computation time and the CV of the RMSE between fruit growth at different topological scales with respect to the M scale, for three tree structures and different friction parameters (*h*). (A) ‘Fuji’; (B) AP-05; (C) AP-10.

Changing topological scale had also a significant impact on fruit growth. In order to ease interpretation, and based on the above fitted values, only results for the best friction parameter values (2 ≤ *h* ≤ 8) are presented. Fruit growth at GU and TBS scales was more correlated to results at M scale than at coarser scales (BR1, FU) (Fig. 5). Generally, in simulations at coarse scales, fruit growth prediction was lower with respect to the M scale. For instance, when running the simulations at FU scale, differences in mean fruit dry weight compared with those obtained at M scale (used in the PEACH model; Allen *et al.*, 2005) increased to 60 % in terms of CV of the RMSE (Fig. 6B).

As expected, multiple fruits belonging to the same coarse-scale component had the same growth (size of spheres in Fig. 3). This was due to the facts that that all fruits at the beginning of the simulation had identical weight and that the C allocated to a coarse-scale component is proportionally divided according to the C demand of individual metamers. As a consequence, the higher the resolution in representing the plant structure (M > GU > TBS > first-order branching ≈ FU), the higher the number of fruits attaining a growth different from all other fruits (Fig. 5). Globally, the lower the friction parameter the lower the range of fruit growth variability, in all the tree structures and at all topological scales (Figs 4C and 5D). The corollary of these observations is that high values of the friction parameter result in a relatively wide range of fruit growth at all scales, but with lower within-tree variability when moving from M to a coarse scale.

### Simplification of the tree structures and computation efficiency

Computation time was found to be a third-order polynomial function of the number of components in the plant (Supplementary Information Fig. 1). The reduction in the number of represented plant components obtained by changing scale from M to FU (down to 0.8 %) in the presented simulations resulted in a gain in computation time of up to four orders of magnitude (down to 0.2 %) (Table 4).

The reduction in computation time associated with the use of coarser scales (Table 4) corresponded to an increased discrepancy (in terms of the CV of the RMSE) in the results obtained between M and other scales (Fig. 6B). Error varied from values close to zero for low friction parameters at GU scale to values up to 60 % for higher friction parameter values and BR1 or GU scales, and was generally higher for higher fiction parameter values. There were, however, minor exceptions to this general behaviour, with low friction parameter values occasionally providing slightly higher discrepancies than higher values (e.g. for *h* = 4 at BR1 scale in the Ap-05 and Ap-10 tree structure, with respect to *h* = 8).

## DISCUSSION

### Multi-scale coherence, impact on predictions and computation time

To our knowledge, MuSCA is the first C-allocation model that allows simulation of C allocation at multiple topological scales within a plant representation. Simulation results revealed that the model was able to produce results that are highly correlated with the M scale, especially when running at GU scale (Fig. 5). As a rule of thumb, the deviation between predictions obtained at M and other scales increased when up-scaling and for increasing friction parameter values (Figs 5 and 6).

Differences in C allocated to fruits at M and other scales are due to two factors. Firstly, after allocation, the C received by the different plant parts belonging to a component is no longer influenced by their individual positions. Thus, the model represents the effects of distances among the components at the coarse scale selected for the simulation, but not among its constituting elements at the finer scale. Second, while the top and basal coordinates of a coarse-scale component are inherited from the finer-scale components present at its extremities, the coordinates of its barycentre are not. Indeed, these are computed as the mean of the coordinates of its components, weighted by their individual lengths. When distances and C flows are calculated, this generates deviations in respect to the M scale.

The deviations between M and other scales were also affected by the specific plant structure (Fig. 5). This is likely related to the non-linearity of the simulated process and its relation to the different discretization of the plant. In trees, the different contributions with respect to the available C of annual organs (leaves and fruits) and the presence of the moderate C sink of woody organs may create contrasting patches in terms of C supplies and C sinks (Fig. 4). When the tree is discretized at different scales, the distances between patches of sources and sinks and their constituting elements are modified. By changing discretization, the set of distances to which the non-linear function (eqn 1) is applied to compute C flows also changes, implying sharp differences in C allocation. Changes are thus related to the geometry of the tree structure as well as to the specific friction parameter, as they determine the spatial domain and the impact of the C-allocation rule. In this regard, further research would be required to explore the impact of commonly applied equations for C allocation (e.g. eqn 1; equations in Balandier *et al.*, 2000; Lescourret *et al.*, 2011) on contrasting tree architectures in terms of distributions of sinks and supplies in relation to their discretization.

Changing topological scale also had a strong impact on computation time. This is an important limiting factor for the simulation of C allocation in complex tree structures, and consequently simulations of tree growth at high spatial detail tend to be limited to relatively young plants (Balandier *et al.*, 2000; Pallas *et al.*, 2016). Computation time in MuSCA closely approximates a third-order polynomial function of the number of represented plant components (Supplementary Information Fig. 1), suggesting the need to reduce plant complexity to simulate plant growth with this type of source–sink model. In this study, the GU scale was able to reproduce values and fruit growth distributions almost identical to M, while saving computation time (Figs 5 and 6). It is possible that the optimal scale of representation may be influenced by tree size. Because of the exponential increase in tree topological complexity with tree age, relatively small variations in C dynamics in early growth stages may have important consequences for the development of the tree structure at later stages. We propose the hypothesis that scales intermediate between M and GU may further decrease the deviations with respect to M scale. We further suggest that a mixed use of high and relatively low topological resolutions for young and older trees, respectively, may optimize prediction accuracy while still allowing simulations on mature trees.

### Testing physiological assumptions

The formalization used in MuSCA to calculate C allocation (eqn 1a) (Balandier *et al.*, 2000) represented the impact of C availability and competition among sinks, such as fruits (Fig. 4), on fruit growth variability.

The RGRs at compartment level were in the lower range of field observations. This is due to the choice of simulation for a partly cloudy day, in which C assimilation could limit growth, resulting in enhanced competition for C (whereas simulations for very sunny days resulted in higher growth and reduced fruit growth variability; data not shown).

In MuSCA, the considered friction parameter (*h*, eqn 1) is empirical and does not correspond to a clear biological process. As in previous studies, this parameter has been estimated by trial and error (Balandier *et al.*, 2000). Nonetheless, the comparison between the simulated and harvested normalized fruit growth distributions suggests that *h* values should be between 4 and 8 (Fig. 4C). This suggests that neither a common assimilate pool (Heuvelink, 1995) of C nor shoot or branch autonomy (Sprugel *et al.*, 1991) adequately accounted for assimilate partitioning within a fruit tree as observed in previous experimental studies (Walcroft *et al.*, 2004; Volpe *et al.*, 2008). Moreover, fluxes from parts of trees with high C supply to parts with a low supply have already been used for modelling within-tree variation in fruit size in peach and apple trees (Lescourret *et al.*, 2011; Pallas *et al.*, 2016). Nevertheless, it has also been observed that the level of shoot or branch autonomy can vary depending on the phenological date, with higher branch autonomy during the summer period compared with winter (Lacointe *et al.*, 2004). Further simulations and analyses with MuSCA performed by dynamically tuning the friction parameter values could help in testing such behaviours.

### Advantages of flexible carbon allocation at multiple scales

By making dynamic use of the MTG (Godin and Caraglio, 1998), the presented model allows us to modify the type of ‘individual entities’ considered in source–sink exchanges within a plant structure on the fly. This is also made possible by formalisms that are equally applicable at all scales (Fig. 2, eqns 2 and 3) and inherit, from fine to coarse scales, the spatial (coordinates of scale boundaries) and extensive (C demands and supplies, up- and down- scaling) properties necessary for the calculation of C flows.

The flexibility related to the multi-scale features of the model provides several opportunities. While the topological scale typically represented in plant models mirrors the interests of a particular group of model users, changing the scale of representation makes the model of practical interest for multiple objectives. As shown in this study, the model can be used to assess the implications of using specific scales. From our results, in most cases and for trees of this size, the GU scale would be a robust alternative to M, while allowing much faster simulations. Comparisons of results among C allocation models could be facilitated by adapting the scale of representation. For instance, the individual fruit growth simulated at the FU scale can be compared with results obtained with QualiTree (Lescourret *et al.*, 2011).

In this study, MuSCA was applied to MTGs derived from the MAppleT architectural model (Costes *et al.*, 2008). However, MTGs from other sources, such as the several models present in the OpenAlea environment, could also be used, given their compliance with certain prerequisites. Also, the level of detail in the description of the plant could be lower since, technically, a representation at M resolution is not strictly necessary. Indeed, in order to run MuSCA, the plant description should simply allow the identification of organ types (via the presence of leaves and fruits), their geometrical sizes (length and radiuses) and their topological connections (succession, branching). This makes the model applicable to tree structures acquired in the field by methods such as the terrestrial laser scanner (TLS), followed by topological reconstruction of the plant (Raumonen *et al.*, 2013; Boudon *et al.*, 2014; Reyes, 2016). In addition, field data collected at various topological scales might still be suitable for model testing. Indeed, by using up- and down-scaling, results simulated at any spatial scale can be brought to the scale at which the data for validation are available.

### Model limitations and further developments

MuSCA still does not represent some important physiological implications of the distribution of sinks along the plant topology. In particular, the influence of individual sinks present in the path between sources and sinks on C flow is not fully described, as in models based on an electric analogy and L-system formalisms (Allen *et al.*, 2005; Cieslak *et al.*, 2011). Indeed, two identical C sinks (D1, D2) present at the same distance from a source (S) will receive from this source an identical amount of C, no matter whether along the path between S and D1 there are stronger or more abundant C sinks than along the path between S and D2. Nevertheless, simple models like ours have the advantage of requiring the calibration of one parameter only compared with these previous approaches.

It is important to remember that, like other C allocation models applicable to large plant structures, MuSCA makes use of an empirical parameter (the friction parameter). In this study we identified a range of possibly sound parameter values, but more appropriate ones could be experimentally estimated from measurements of the sap flow through isotopically labelled C (Hansen, 1967), as well as measurements of fruit growth in simple tree structures with modified fruit loads.

The MuSCA model could be further refined by using MTG properties to increase computational efficiency without losing prediction accuracy. For instance, when running at coarse scale, the direction of origin of C assimilates might be used to account for distance also within the boundaries of the coarse-scale component.

The creation of new metamers should be included in order to allow the simulation of plant growth during elongation and growth periods (on non-fixed structures). In addition, given the influence of pruning on the distribution and emergence of C sinks, the implementation of a module describing reactions to this management practice (e.g. DeJong *et al.*, 2012) would significantly extend the applicability of the model to a broader range of real cases. Further, a more detailed description of the root would be the starting point to investigate water and nutrient limitations at the soil interface.

Regarding the use of MuSCA in the larger context, its generality can ease its adaptation to different species. In addition, the modular implementation of MuSCA in the OpenAlea environment facilitates integration with other, previously developed models, as was the case for the connection with the MAppleT (Costes *et al.*, 2008) and the RATP models (Sinoquet *et al.*, 2001).

### Conclusions

In this study we present MuSCA, to our knowledge the first C-allocation model allowing the simulation of C allocation at multiple topological scales of representation of the plant. The presented model provides topologically based methods to re-interpret/simplify the topological scale at which the process of C allocation is simulated. The simulations revealed a major impact of the topological scale, used to discretize C sources and sinks, on the predicted C allocation, even when other C allocation rules (equation for C allocation and friction parameter) were kept constant. The model can be used to identify which degree of simplification is acceptable for the representation of plant structures without compromising accuracy in the computation of C allocation. It can be used on large plants, being aware of the trade-offs in terms of computation time and prediction accuracy. In addition, the flexible representation of the plant topology facilitates matching the needs of different users, while using the same model. For instance, a relatively coarse scale (e.g. branch) could be more suitable for a farmer interested in fruit thinning than a fine one (e.g. metamer), which could be the target of a modeller interested in investigating the local drivers of individual fruit size variability.

## SUPPLEMENTARY DATA

Supplementary data are available at Annals of Botany online and consist of the following. File S1: Inputs of the MuSCA model and Input tree structures. Figure S1: computation time in respect of the number of represented structure components.

mcz122_suppl_Supplementary_Figure

mcz122_suppl_Supplementary_File
