# Supplementary figures and images for: MuSCA: a multi-scale source–sink carbon allocation model to explore carbon allocation in plants. An application to static apple tree structures
Source: Ann Bot. 2019 Oct 23;126(4):571–85. doi: 10.1093/aob/mcz122 (PMC7489079; doi:10.1093/aob/mcz122)

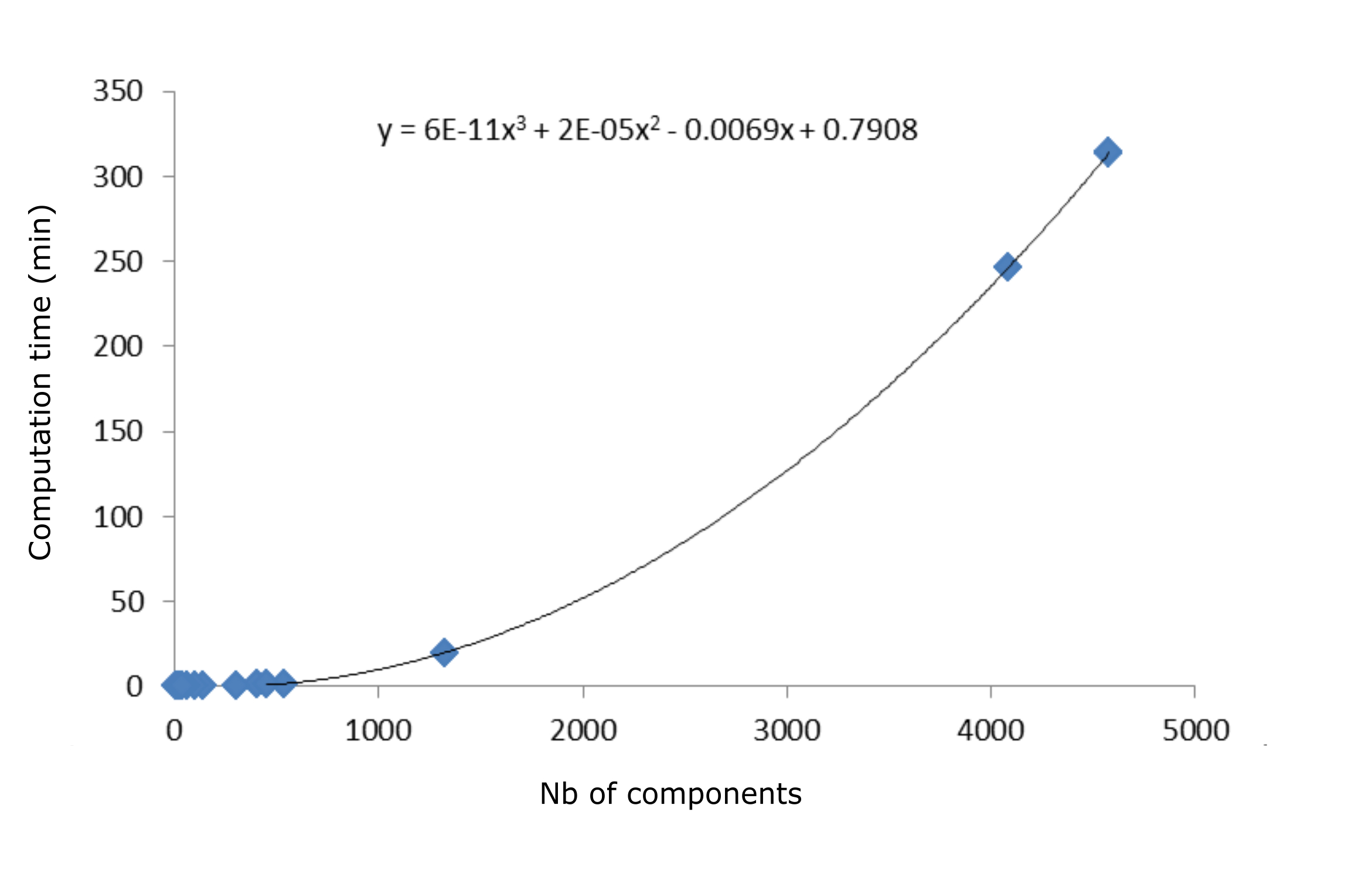

Supplement: mcz122_suppl_Supplementary_Figure [file mcz122_suppl_supplementary_figure.jpeg]
