# Supplementary material for: MuSCA: a multi-scale source–sink carbon allocation model to explore carbon allocation in plants. An application to static apple tree structures
Source: Ann Bot. 2019 Oct 23;126(4):571–85. doi: 10.1093/aob/mcz122 (PMC7489079; doi:10.1093/aob/mcz122)
Supplement: mcz122_suppl_Supplementary_File [file mcz122_suppl_supplementary_file.docx]

# Supplementary Information

## Supplementary Information: Inputs of the MuSCA model.

| Variable | Description | Unit |
| --- | --- | --- |
| *Multi-scale Tree Graph description of a plant (.mtg)* | |  |
| *XX, YY, ZZ* | Coordinates of the centre of the top of a plant component | m |
| *radius* | tree component apical radius | m |
| *leaf_area* | leaf area surface | m^^2^ |
| *fruit* | fresh fruit biomass | Kg |
| *observation* | organ type: trunk, other types | - |
|  |  |  |
| Topological connector | “>” for succession; “+” for branching | - |
| Scale boundary | “/” for boundary across scales |  |
|  |  |  |
| *Meteorological file for the RATP model (.mto)* | |  |
| day | day of the year | day |
| hour | index of the current semi-hour from midnight | 30 min |
| PARglob | global incident Photosynthetically Active Radiation | W m^^-2^ |
| PARdif | diffused Photosynthetically Active Radiation | W m^^-2^ |
| NIRglob | global incident Near Infrared Radiation | W m^^-2^ |
| NIRdif | diffused Near Infrared Radiation | W m^^-2^ |
| Ratmos | Total incoming radiation | W m^^-2^ |
| Tsol | mean soil temperature | °C |
| Tair | mean air temperature | °C |
| Eair | H2O partial pressure | Pa |
| CO2air | CO2 partial pressure | Pa |
| Wind | wind speed above tree | m s^^-1^ |

## Supplementary Information: Input tree structures

In the current application, input tree structures were MTGs produced by the MAppleT model (Costes *et al.* 2008). MAppleT includes (i) Markov models for simulating annual shoot branching and successive growth across years, (ii) a biomechanical model simulating the change in branch form over time, and (iii) a modified pipe-model to estimate axis radial growth. The leaf area and internode length depend on their rank along the shoot (Da Silva, Han, and Costes 2014). The MTGs output produced by MappleT are apple trees structures at a certain stage of development (given date of the year), represented at two scales, the growth unit and metamer. Metamer attributes are its apical and basal 3D coordinates, stem diameter, leaf area and fruit weight (if present).

At the beginning of the simulation, the model uses the geometrical description of the plant and some species specific parameters (Reyes *et al.* 2016, in-field observations) to estimate the initial dry weight of each plant component at the metamer scale (see Supplementary Information: Inputs of the MuSCA model). In particular: dry weight of old wood and vegetative internodes are computed as functions of their geometrical description (lengths and radiuses), and of a constant (wood density) or a function of thermal time, respectively; fruits dry weight is calculated from fresh weight and a constant (dry to fresh fruit dry weight ratio); leaf dry weight is calculated from surface and a constant (per unit surface leaf dry mass) (Table 1). Dry mass of individual internodes is calculated as follows: volumes of internodes are first computed as truncated cones, summed up to provide the volume of individual shoots and stored at a “current year shoot” coarse scale. The length of current year shoots is then obtained by summing the lengths of their internodes, and used to estimate shoot biomass by means of a thermal-time dependent allometric relationship (Table 1) (Reyes *et al.* 2016). Finally, the dry biomass of individual internodes is calculated as their individual volumetric fraction in the shoot to which they belong, multiplied by the shoot dry biomass.

Regarding the root, a rough representation of this compartment was added to the plant. A root mass, defined as proportional to the total mass of the current year shoots, is added to the root metamer following the shoot/root functional balance assumption (Davidson 1969; Grechi *et al.* 2007). The length of the roots is represented as equal to half the average distance between the soil and the vegetative shoots. The root basal coordinates are thus defined equal to the tree basal coordinates, except for the vertical (z axis) component, to which the calculated distance is subtracted (downward translation).
